# Supplementary figures and images for: Cryo-EM structure of the fully-loaded asymmetric anthrax lethal toxin in its heptameric pre-pore state
Source: PLoS Pathog. 2020 Aug 18;16(8):e1008530. doi: 10.1371/journal.ppat.1008530 (PMC7462287; doi:10.1371/journal.ppat.1008530)

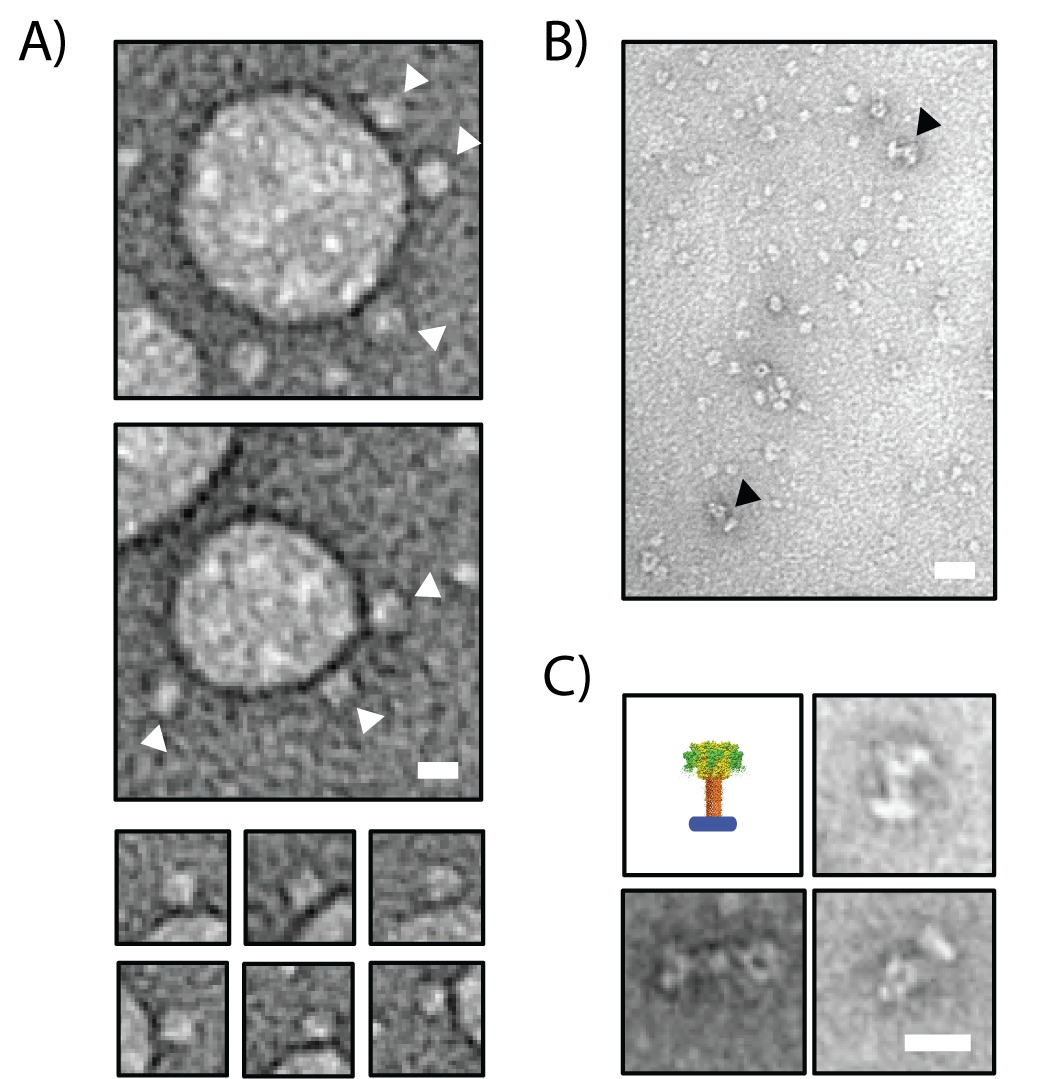

Supplement: S1 Fig — (A) Representative negatively stained electron micrograph areas of PA7 reconstituted into POPC liposomes (top panels), with individual inserted particles highlighted by white arrowheads. Selection of inserted particles in smaller lipid vesicles (bottom panel). Scale bar: 15 nm. Particles are clearly accumulated at lipid membranes. (B) Representative negatively stained electron micrograph area of PA7 reconstituted in lipid nanodiscs (MSP1D1), with individual inserted particles highlighted by black arrowheads. Scale bar: 20 nm (C) Model of PA7 complexes inserted into lipid nanodiscs with additional examples of individual particles after reconstitution (same nanodiscs as in B). Scale bar: 20 nm. (TIF) [file ppat.1008530.s001.tif]

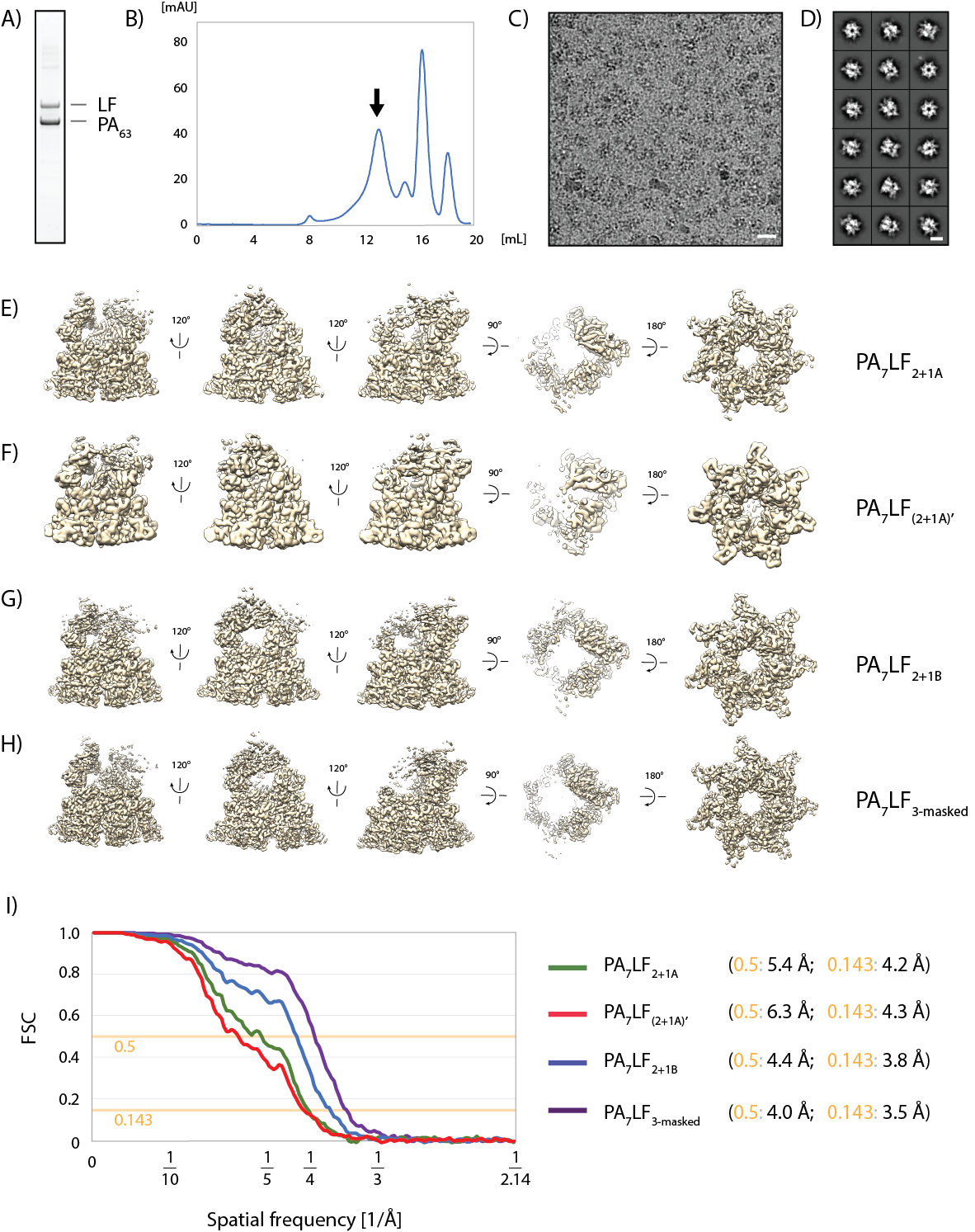

Supplement: S2 Fig — (A) Coomassie-stained SDS-PAGE of purified PA7LF3 complex. (B) Size exclusion chromatography profile of the PA7LF3 complex using a Superdex 200 column. Sample fraction used for cryo-EM studies is indicated by black arrow. (C) Representative digital micrograph area of vitrified PA7LF3 complex. Scale bar: 20 nm. (D) Representative 2-D class averages corresponding to C. Scale bar: 10 nm. (E-H) Rotated views of the 3-D reconstruction of PA7LF2+1A (E), PA7LF(2+1A)’ (F), PA7LF2+1B (G), and PA7LF3-masked (H), respectively. (I) FSC curves between two independently refined half-maps of PA7LF2+1A (green), PA7LF(2+1A)’ (red), PA7LF2+1B (blue) and PA7LF3-masked (purple). (TIF) [file ppat.1008530.s002.tif]

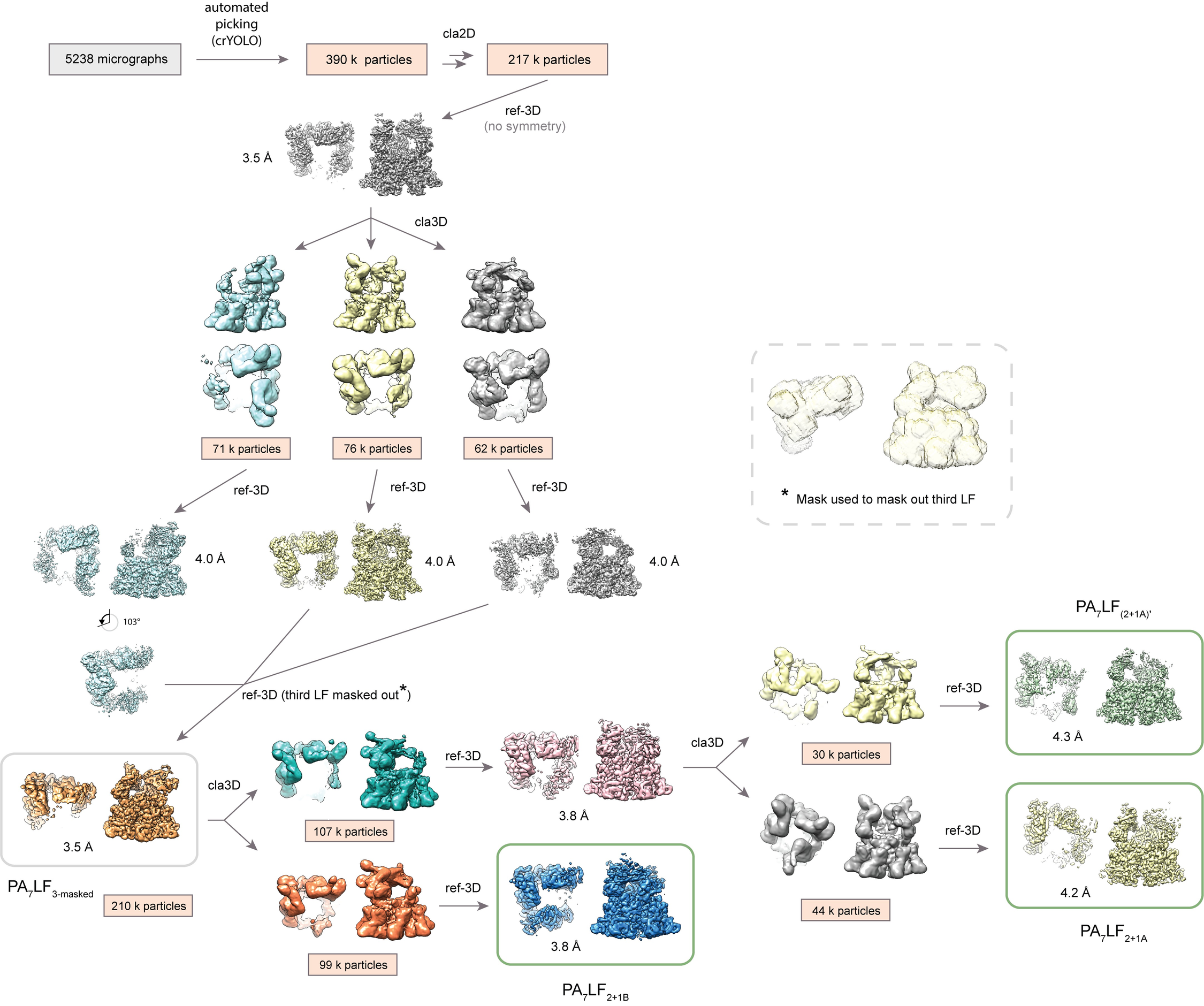

Supplement: S3 Fig — The single particle processing workflow is shown that included multiple 3-D classification steps as well as rotation of individual classes (indicated by rotation symbol). Number of particles in each class is provided as orange box below the respective structure and the obtained resolution of the map after 3-D refinement is indicated. For each structure a top and side view is shown (in top views PA7 density is partially clipped to focus on the bound LFs). Mask for masking out third LF is provided in dashed box. Final electron density maps are highlighted by green boxes. Abbreviations: cla3D – 3-D classification, cla2D – 2-D classification, ref-3D – 3-D refinement. (TIF) [file ppat.1008530.s003.tif]

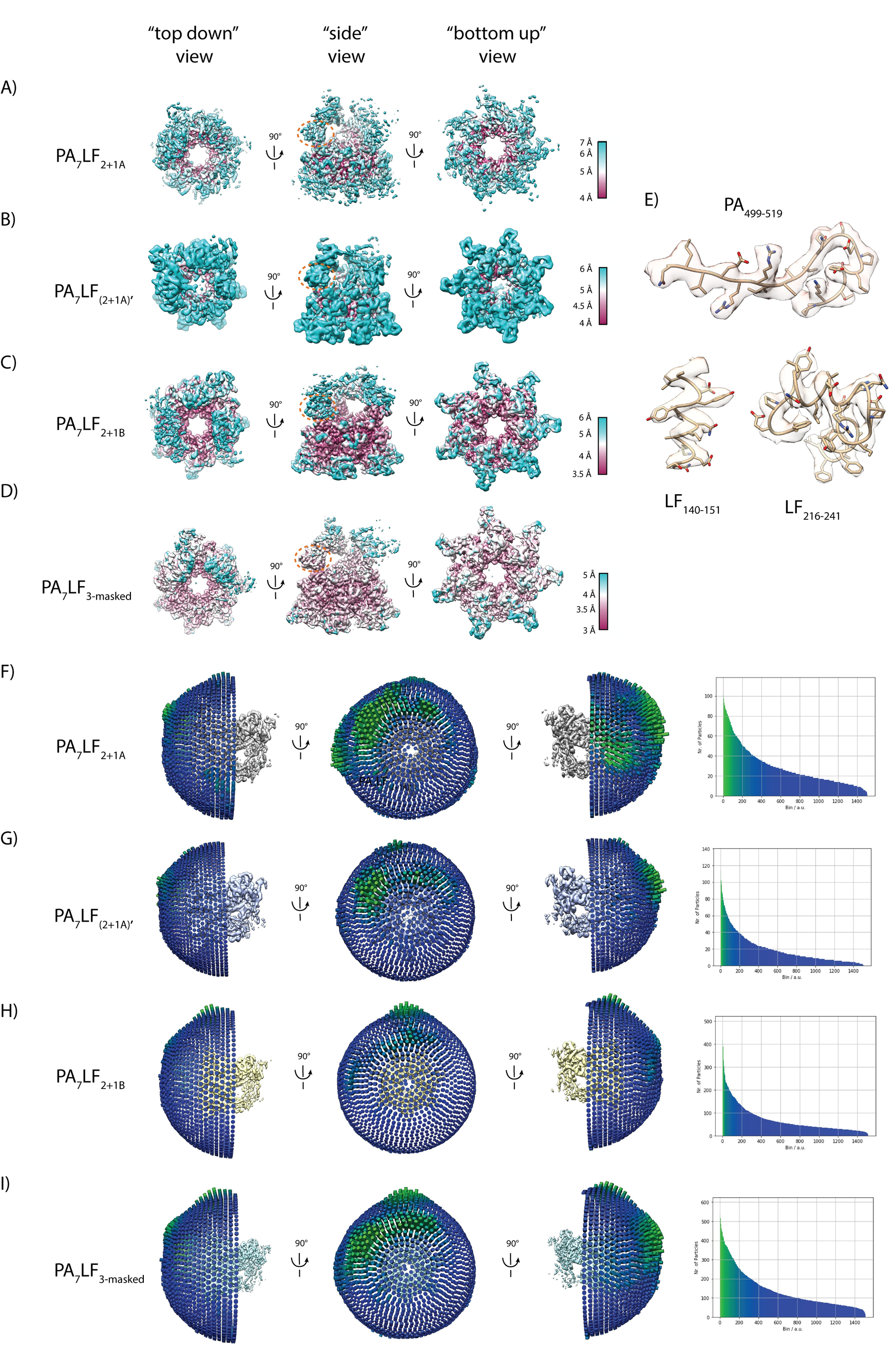

Supplement: S4 Fig — (A-D) Rotated views of the reconstructions, PA7LF2+1A (A), PA7LF(2+1A)’ (B), PA7LF2+1B (C), and PA7LF3-masked (D), respectively, colored by local resolution. Corresponding color key of local resolution is provided on the right. The position of the N-terminal domain of 1LF is indicated by a dashed orange ellipse for orientation. (E) Selected examples of side chain densities corresponding to PA and LF with atomic models fitted. (F) Rotated views of the 3-D angular distribution plot for the PA7LF2+1A reconstruction, in which the relative height of bars represents the number of containing particles. Corresponding 2-D histogram is shown on the right. (G-I) Same as in F for PA7LF(2+1A)’ (G), PA7LF2+1B (H), and PA7LF3-masked (I). (TIF) [file ppat.1008530.s004.tif]

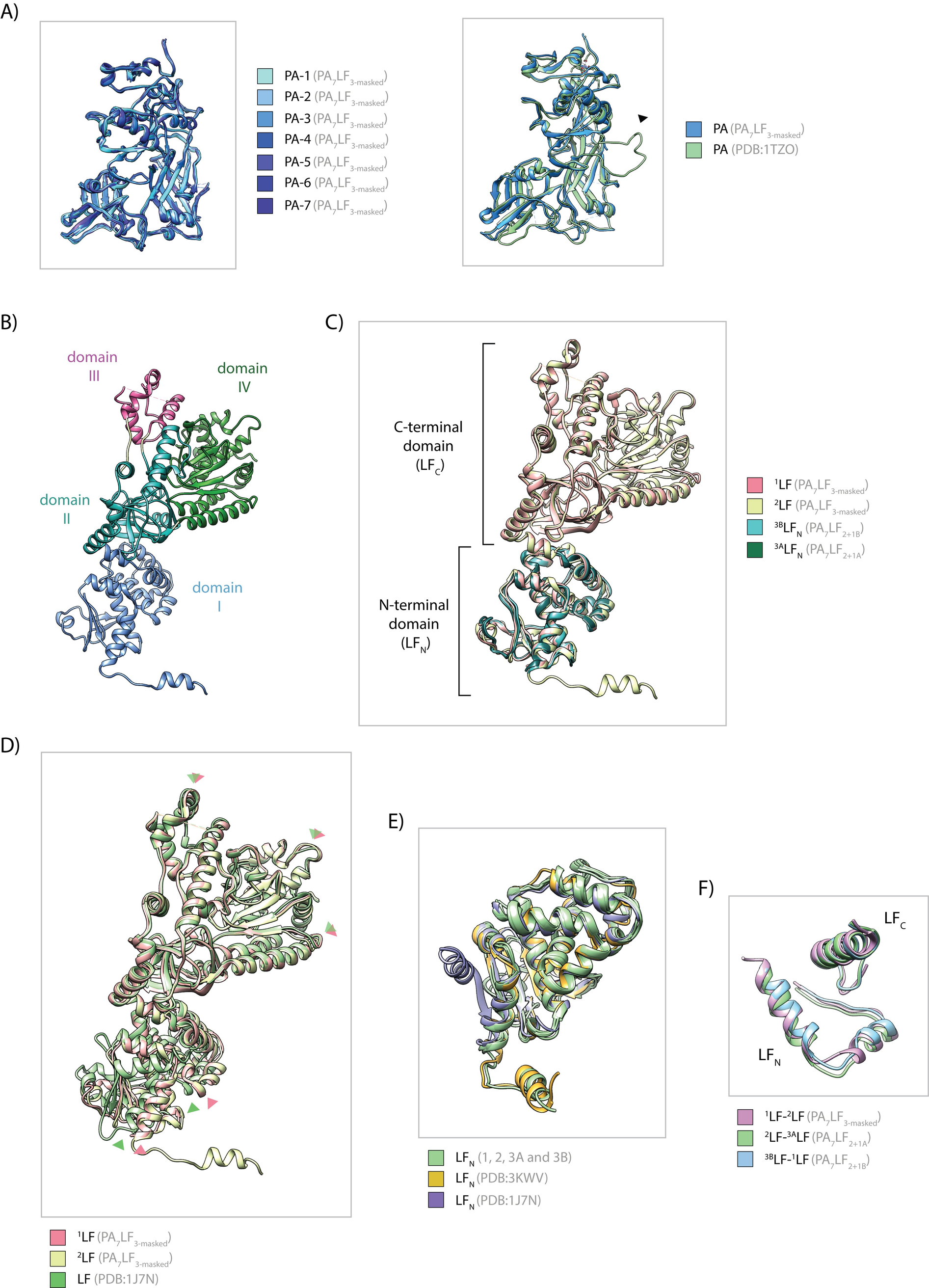

Supplement: S5 Fig — (A) Superposition of the seven PA protomers in PA7LF3, which are colored in different blue hues (left panel), and a single PA subunit (blue) with the known crystal structure (PDB: 1TZO, green, right panel). Loop region 2β2-2β3 (residues 300–323), resolved only in the crystal structure, is highlighted by a black arrowhead. (B) Domain organization of LF with individual domains highlighted by different colors. (C) Superposition of individual LFs in the PA7LF3 structures with 1LF in pink, 2LF in gold, 3BLFN in cyan and 3ALFN in dark green. (D) Superposition of 1LF (pink), 2LF (gold) and unbound LF (PDB: 1J7N, green), aligned via their C-terminal domain. Green and red arrows indicate similar positions in 1LF and unbound LF (PDB:1J7N), respectively. Comparison reveals that the C-terminal domain is rotated respective to the N-terminal domain in the PA7LF3 structures. (E) Superposition of the N-terminal domain of the three LFs in PA7LF3 (green), of LF in the “open” conformation in PA8LF4 (PDB: 3KWV, dark yellow) and of unbound LF in the “closed” conformation (PDB: 1J7N, purple). (F) Superposition of the three LF-LF interfaces with 1LF-2LF in pink, 2LF-3ALF in green and 3BLF-1LF in blue. (TIF) [file ppat.1008530.s005.tif]

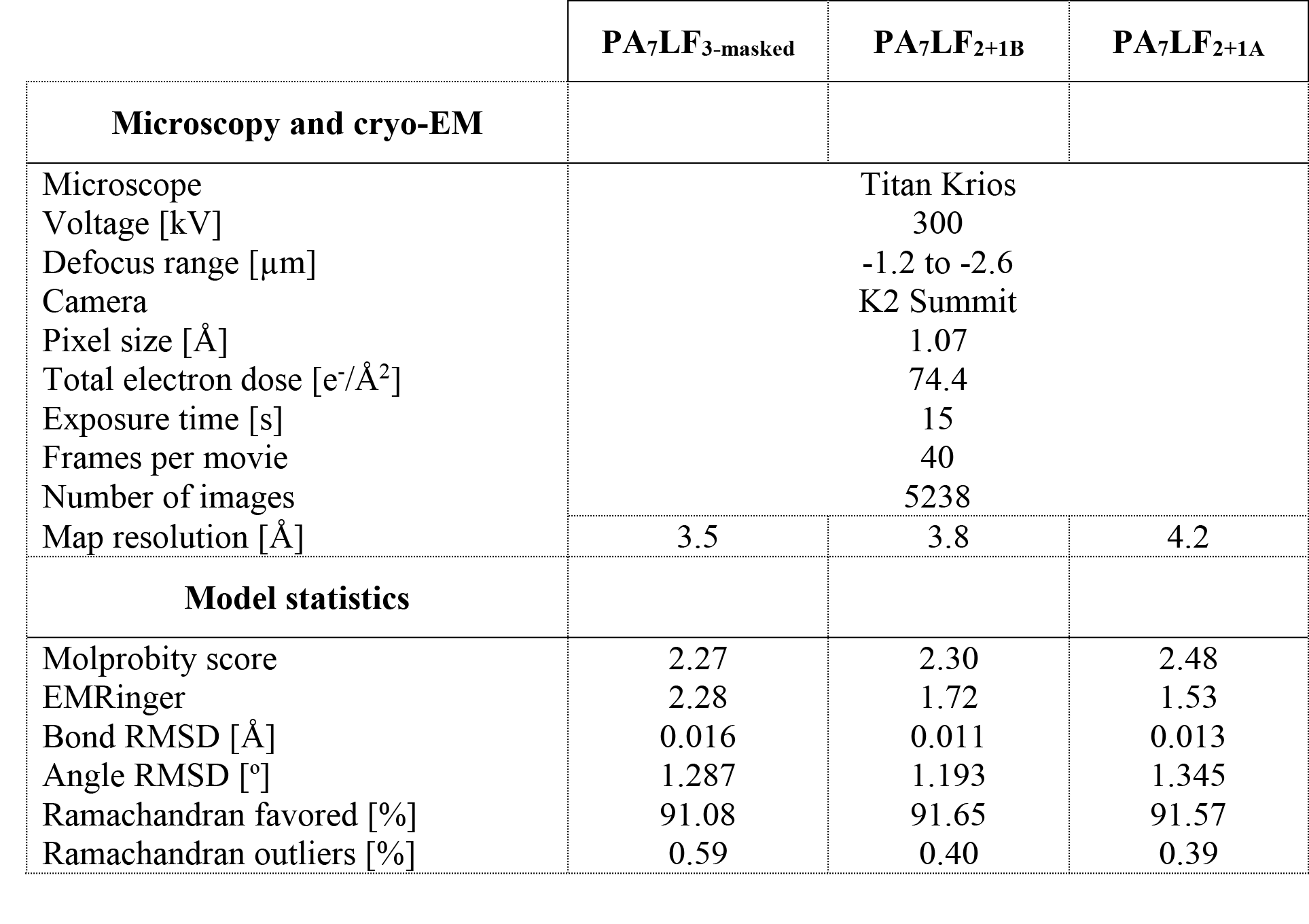

Supplement: S1 Table — (TIF) [file ppat.1008530.s006.tif]
