# Supplementary material for: Cryo-EM structure of the fully-loaded asymmetric anthrax lethal toxin in its heptameric pre-pore state
Source: PLoS Pathog. 2020 Aug 18;16(8):e1008530. doi: 10.1371/journal.ppat.1008530 (PMC7462287; doi:10.1371/journal.ppat.1008530)
Supplement: Attachment — Submitted filename: Review_PLOSPathogens.docx [file ppat.1008530.s009.docx]

PLOS Pathogens

Cryo-EM structure of the fully-loaded asymmetric anthrax lethal toxin in its heptameric pre-pore state

**Summary**

The authors show the structures of pre-pore PA7 fully loaded with three LF subunits. The Structures for the individual components (PA, PA7, and LF) have been solved previously as well as a crystal structure of PA8LF4. However, these results show different conformations of LF bound to PA7 which have not been observed previously. The authors describe the interfaces between LF in different conformations with PA and also interfaces between LF subunits. They note that one subunit (2LF) is bound in an “open” conformation which results in a unique interaction between an N terminal alpha helix of LF with PA as well as a disordered C terminus of LF which may promote translocation of the substrate in the unfolded state. The other F subunits are described as being in an “intermediate” state between the “open” state observed in 2LF and the “closed” state observed in the crystal structure of unbound LF (where “closed” or “open” describe position of the N-terminal alpha helix relative to the rest of the protein). The authors then speculate about the order of LF subunits translocation by PA7 as well as the order of LF subunit binding to PA7.

The structures in this work are of high quality and well-done. The manuscript is easy to read. The symmetry mismatch between the pre-pore PA7 and the LF subunits is interesting and the observation of a previously unidentified structural conformations the LF subunit is novel. The only major issue is the suggested ordering of LF binding to the PA7 ring. The cryo-EM data provides no kinetic or timing information and the authors discussions about favoring one model over the other (of order of binding) is not strong. Is there any additionally (already published) kinetic data or other models already proposed in the field that could bolster this part of the discussion. Without any other information than just the EM structures the discussion of the model will need to be modified. Especially in this time of COVID-19 shutdowns (but even if all the labs were still open) – I feel that this paper is strong enough to be published without going back to the bench to do any experiments to address the timing and/or order of binding. The focus of this paper is the presentation of a structure that has not been seen before and this can definitely be used to suggest new models. However, the suggestion of the timing/order of binding and translocation needs to either be presented as the possibility of multiple models and/or include additional arguments from published literature.

**Big Picture Concerns/Questions - Discussion**

- The numbering of the LFs and binding sites doesn’t quite make sense, especially in the context of the discussion about the order in which LFs might be translocated. In the (2+1A) structures, 2LF is in the middle, making contact with 1LF on one side and 3LF on the other. In the (2+1B) structure, 2LF only contacts 1LF. The authors propose that the possibilities for the order of translocation of LFs “exclude a chain reaction. However the first scenario proposed is that 2LF is translocated first followed by 1LF or 3LF. In the (2+1B) conformation, translocation of 2LF, followed by 1LF, and then 3LF would be a "chain reaction.” Similarly, for the second proposed scenario, translocating 3lF, then 1LF, then 2LF would be a “chain reaction.”
- There are no experimental data in this paper for the order of translocation LF by PA7 or for the order of LF binding to PA7. There is some reasoning for the suggested order of translocation provided by the authors. There is no reasoning given for the proposed binding order. The model shown in Figure 6 is not supported by data. Can the authors rework the figure and/or add previous data from the literature that would support various models?

**Other questions/comments (can be addressed by written responses)**

- Line 114 & Figure S3 – I don’t understand what is meant by the mixture of assemblies being “partly unassignable”? All three assignments are shown in this workflow. Does this statement just refer to some particles not being included in one of the final classes?
- Why was it necessary to use a filtered crystal structure for the final refinement?
- The position of subunits in Figure 2 is confusing. In 2A, the inset close-up seems to be oriented opposite the zoomed-out image. (PA is on the left in the full complex image but on the right in the close-up image.) If that’s the case, please indicate the direction and degree of rotation. It’s also not clear where the second interface shown in 2B is located relative to the first interface shown in 2A. Furthermore the interface in 2A is described in the text as KF interacting with two adjacent PAs. From the inset, this interaction appears to be primarily with PA and only peripherally with PA’.
- Line 161 include citation or PDB for unbound LF to make clear that the unbound LF structure used for comparison here is from previous work and not these structures.
- The section below (line 175-179) is not clear. What is meant by the interaction, the additional interface, the main interface?
  - However, the position of the interaction differs from the additional interface, that has been proposed by Fabre et al. (34). At the main 2LF-1LF interface, the helix-loop region (residues 572-579) of the first lethal factor (1LF) forms a relatively small
- In line 240-243, what is “it”? PA? The alpha-clamp? ClpA/Hsp100?
  - The current theory is that it first stabilizes unfolding intermediates, and introduces mechanical strain before the unfolded structure is fed further down the central pore (21). In this way it would facilitate the rapid unfolding of the entire 2LF molecule upon transition into the pore state.
- Is the comparison of PA7 translocation activity to ClpA/Hsp100 translocation activity relevent? PA7 translocates substrates across a membrane using proton motive force. ClpA translocates cytosolic proteins, using the energy from ATP binding and hydrolysis, into the protease ClpP. In ClpA, the movement of the P loop during ATP binding and hydrolysis physically pushes the substrate the pore of ClpA. The mechanism of PA7 translocation must be quite different.

**Small recommended edits**

- Supplemental Figure 4 A-D would be easier to read with additional labels. For example could you label the view where the reader is looking “top down” at LF? Where is the “central N terminal domain which is resolved best”?
- In Figure 2B, the panel for ^1^LF has gold text reading ^2^LF overlaid with the pink structure.
- It seems that the unbound LF is the “closed” conformation. If that’s the case, please state it clearly. Consider using both labels on figures.
- Consider adding labels for “open” (gold), “closed/unbound”(blue), and intermediate (red) conformations in Figure 4A.
- Line166: indicate which LF is referenced at the end of this line
  - A steric clash between the loop region (residues 576-579) of 1LF and a1-b1 of 2LF (Fig. 4A) prevents the N-terminal a-helix of LF from remaining in the “closed” conformation…
