# Supplementary material for: Cryo-EM structure of the fully-loaded asymmetric anthrax lethal toxin in its heptameric pre-pore state
Source: PLoS Pathog. 2020 Aug 18;16(8):e1008530. doi: 10.1371/journal.ppat.1008530 (PMC7462287; doi:10.1371/journal.ppat.1008530)
Supplement: Attachment — Submitted filename: Antoni_etal_response_reviewers_comments.docx [file ppat.1008530.s010.docx]

We thank the reviewers for their constructive and positive feedback, which aided us to further improve the manuscript. Major modifications of the manuscript are highlighted in yellow. Below we include our detailed response to each point.

**Part I - Summary**
Please use this section to discuss strengths/weaknesses of study, novelty/significance, general execution and scholarship.

**Reviewer #1**: The authors show the structures of pre-pore PA7 fully loaded with three LF subunits. The Structures for the individual components (PA, PA7, and LF) have been solved previously as well as a crystal structure of PA8LF4. However, these results show different conformations of LF bound to PA7 which have not been observed previously. The authors describe the interfaces between LF in different conformations with PA and also interfaces between LF subunits. They note that one subunit (2LF) is bound in an “open” conformation which results in a unique interaction between an N terminal alpha helix of LF with PA as well as a disordered C terminus of LF which may promote translocation of the substrate in the unfolded state. The other F subunits are described as being in an “intermediate” state between the “open” state observed in 2LF and the “closed” state observed in the crystal structure of unbound LF (where “closed” or “open” describe position of the N-terminal alpha helix relative to the rest of the protein). The authors then speculate about the order of LF subunits translocation by PA7 as well as the order of LF subunit binding to PA7.

The structures in this work are of high quality and well-done. The manuscript is easy to read. The symmetry mismatch between the pre-pore PA7 and the LF subunits is interesting and the observation of a previously unidentified structural conformations the LF subunit is novel. The only major issue is the suggested ordering of LF binding to the PA7 ring. The cryo-EM data provides no kinetic or timing information and the authors discussions about favoring one model over the other (of order of binding) is not strong. Is there any additionally (already published) kinetic data or other models already proposed in the field that could bolster this part of the discussion. Without any other information than just the EM structures the discussion of the model will need to be modified. Especially in this time of COVID-19 shutdowns (but even if all the labs were still open) – I feel that this paper is strong enough to be published without going back to the bench to do any experiments to address the timing and/or order of binding. The focus of this paper is the presentation of a structure that has not been seen before and this can definitely be used to suggest new models. However, the suggestion of the timing/order of binding and translocation needs to either be presented as the possibility of multiple models and/or include additional arguments from published literature.

**Reviewer #2**: The manuscript by Antoni and collaborators presents the structure of the anthrax toxin. This complex is constituted of three components: the protective antigen (PA) who forms a multimeric complex that attaches to the TEM8 receptor and two cytotoxic subunits, lethal factor (LF) and the edema factor (EF) which are further translocated into the cytoplasm. The cipher for the geometric assembly of three copies of LF on top of seven copies of PAs was a structural prize followed by many groups over almost two decades.
The present study offers a clear description of the possible distributions of the LFs on the PA ring by calculating a series of cryo-EM maps obtained by sequential 3D classification. Their higher resolution structure shows the LFs arranged in a continuous chain of head-to-tail interactions. However, each of the LF adopt a different conformations which leads the authors to propose a model for the formation of the LFs arrangement and for their translocation.
The manuscript is well constructed, the figures are elegant, clear and informative. I am convinced that the article will be of special interest to readers of PLoS Pathogens and I enthusiastically suggest its publication.

**Reviewer #3**: This manuscript by Antoni and co-workers use high resolution cryo-EM to study anthrax toxin action on host cells. In particular, the authors seek to gain insights into recruitment of lethal factor and edema factor to the heptameric PA pre-pore and subsequent order of translocation.

The author’s work confirm previous findings that three LFs interact with the heptameric PA to form the loaded pre-pore. However, details of these interactions have been limited due to lower resolution structures. In this study, the authors present higher resolution PA-LF complexes, which offer additional details by which to refine the model of LF and EF recruitment to the PA pre-pore.

The studies described in the present study build upon this previous work by providing higher resolution details. Specifically, although the three LF monomers form a continuous chain of head-to-tail “chain” interactions of with the heptameric PA pre-pore state (PA7LF3), the fate of the three monomers differ, in that while all three monomers are not in the “closed “state, one of the three LF monomers adopts an open conformation while interacting with the alpha clamp of PA, while the other two monomers are in what the authors refer to as an “intermediate” state. Because the third LF can bind the PA7-LF2 complex in two fundamentally different ways, the authors appropriately indicate that they cannot predict the translocation order of monomer

Based on the structures the authors propose a model that differs slightly than that recently proposed by another group, which had predicted that LF translocation occurs by a “chain-reaction” mechanism. Here the authors suggest an alternative to the “chain-reaction” model, and predict that, while the first LF to bind the PA7 complex is likely to be the first translocated substrate, that either the second or third LF could be translocated next, perhaps on differences in the binding orientation of the final recruited LF.

Strengths of the manuscript include the importance of the topic, the high quality of the work, and the well written text. Potential issues include similarities to recently published work from another group, which raises questions about the extent to which the results advance the field.

We want to thank all reviewers for their very positive and constructive comments. Below we include the detailed response to each point raised by the reviewers. The changes in the manuscript are highlighted in yellow.

**Part II – Major Issues: Key Experiments Required for Acceptance**
Please use this section to detail the key new experiments or modifications of existing experiments that should be absolutely required to validate study conclusions.

Generally, there should be no more than 3 such required experiments or major modifications for a "Major Revision" recommendation. If more than 3 experiments are necessary to validate the study conclusions, then you are encouraged to recommend "Reject".


**Reviewer #1**: (No Response)

**Reviewer #2**: None

**Reviewer #3**: (No Response)

**Part III – Minor Issues: Editorial and Data Presentation Modifications**
Please use this section for editorial suggestions as well as relatively minor modifications of existing data that would enhance clarity.


**Reviewer #1**:

**Big Picture Concerns/Questions – Discussion**

- The numbering of the LFs and binding sites doesn’t quite make sense, especially in the context of the discussion about the order in which LFs might be translocated. In the (2+1A) structures, 2LF is in the middle, making contact with 1LF on one side and 3LF on the other. In the (2+1B) structure, 2LF only contacts 1LF.

We admit that the naming or numbering of the LFs was not an easy task and alternatives are indeed possible. The rationale behind our naming is based on the local resolution of the individual LFs in our structures. All three structures share a common core, consisting of PA_7_ decorated with the red- and gold-colored LFs. The best resolved LF (red) was named ^1^LF, followed by ^2^LF (gold) and ^3^LF (cyan). A break in the order of the three LFs is inevitable as a consequence of the different assemblies that exist due to the two alternative binding position for the third LF. We added an explanatory statement about the naming in the revised manuscript.

- The authors propose that the possibilities for the order of translocation of LFs “exclude a chain reaction. However the first scenario proposed is that 2LF is translocated first followed by 1LF or 3LF. In the (2+1B) conformation, translocation of 2LF, followed by 1LF, and then 3LF would be a "chain reaction.” Similarly, for the second proposed scenario, translocating 3lF, then 1LF, then 2LF would be a “chain reaction.”

This is a valid point. We revised this part of the discussion.

- There are no experimental data in this paper for the order of translocation LF by PA7 or for the order of LF binding to PA7. There is some reasoning for the suggested order of translocation provided by the authors. There is no reasoning given for the proposed binding order. The model shown in Figure 6 is not supported by data. Can the authors rework the figure and/or add previous data from the literature that would support various models?

Reviewer #1 is right and we changed our model figure (Fig. 6) accordingly. In the revised figure, panels are either supported by our structural data or alternative scenarios are shown.

**Other questions/comments (can be addressed by written responses)**

- Line 114 & Figure S3 – I don’t understand what is meant by the mixture of assemblies being “partly unassignable”? All three assignments are shown in this workflow. Does this statement just refer to some particles not being included in one of the final classes?

This statement refers to the initial 3D reconstruction with all 217k particles (3.5 Å), which is shown in grey at the very top of Figure S3. It represents a mixture of the (2+1A), (2+1A)’ and (2+1B) states, that were only properly classified in the subsequent 3D sorting steps. In addition, this preliminary map contained a subset of particles that required a 103° rotation in order to align the commonly shared core, consisting of PA_7_, ^1^LF and ^2^LF. Hence, this structure did not allow us to accurately assign the LFs as this initial map represents a superposition of different LFs.

We rephrased the corresponding sentence in the manuscript to make this point clearer.

- Why was it necessary to use a filtered crystal structure for the final refinement?

We apologize if this was not stated more clearly, but the filtered density based on the crystal structure was only used in the initial refinement. Here, the converted electron density map was filtered to reduce model bias, as we cannot ensure that our structure might differ from the used PDB model.

In all 3D refinements that followed the 3D sorting steps, reference maps based on either the 3D sorting classes or from previous refinements served as starting model.

We added this part to the material and method section.

- The position of subunits in Figure 2 is confusing. In 2A, the inset close-up seems to be oriented opposite the zoomed-out image. (PA is on the left in the full complex image but on the right in the close-up image.) If that’s the case, please indicate the direction and degree of rotation. It’s also not clear where the second interface shown in 2B is located relative to the first interface shown in 2A.

We changed the orientation of the overview structure (left panel) so that it matches with the close-up (right panel). We also indicated the position of the second interface (close-up in Fig. 2B) in the overview structure in Fig. 2A.

- Furthermore the interface in 2A is described in the text as KF interacting with two adjacent PAs. From the inset, this interaction appears to be primarily with PA and only peripherally with PA’.

We have clarified this point in the revised manuscript.

- Line 161 include citation or PDB for unbound LF to make clear that the unbound LF structure used for comparison here is from previous work and not these structures.

We have added the citation.

- The section below (line 175-179) is not clear. What is meant by the interaction, the additional interface, the main interface?
  - However, the position of the interaction differs from the additional interface, that has been proposed by Fabre et al. (34). At the main 2LF-1LF interface, the helix-loop region (residues 572-579) of the first lethal factor (1LF) forms a relatively small

We have revised this section of the manuscript.

- In line 240-243, what is “it”? PA? The alpha-clamp? ClpA/Hsp100?
  - The current theory is that it first stabilizes unfolding intermediates, and introduces mechanical strain before the unfolded structure is fed further down the central pore (21). In this way it would facilitate the rapid unfolding of the entire 2LF molecule upon transition into the pore state.

We have rephrased these sentences to enhance clarity.

- Is the comparison of PA7 translocation activity to ClpA/Hsp100 translocation activity relevent? PA7 translocates substrates across a membrane using proton motive force. ClpA translocates cytosolic proteins, using the energy from ATP binding and hydrolysis, into the protease ClpP. In ClpA, the movement of the P loop during ATP binding and hydrolysis physically pushes the substrate the pore of ClpA. The mechanism of PA7 translocation must be quite different.

We agree with the reviewer and omitted the comparison with ClpA/Hsp100 in the revised discussion.

**Small recommended edits**

- Supplemental Figure 4 A-D would be easier to read with additional labels. For example could you label the view where the reader is looking “top down” at LF? Where is the “central N terminal domain which is resolved best”?

We added the labels and indicated the position of the N-terminal domain of ^1^LF for orientation.

- In Figure 2B, the panel for ^1^LF has gold text reading ^2^LF overlaid with the pink structure.

The additional label was removed.

- It seems that the unbound LF is the “closed” conformation. If that’s the case, please state it clearly. Consider using both labels on figures.

We added both labels to the figures 3 and 4.

- Consider adding labels for “open” (gold), “closed/unbound”(blue), and intermediate (red) conformations in Figure 4A.

We added the labels in Figure 4A.

- Line166: indicate which LF is referenced at the end of this line
  - A steric clash between the loop region (residues 576-579) of 1LF and a1-b1 of 2LF (Fig. 4A) prevents the N-terminal a-helix of LF from remaining in the “closed” conformation…

We named the LF in the revised sentence.

**Reviewer #2**: Minor points:

• line 275 and others: space between the number and symbol

Done.

• line 304: mention octyl glucoside while introducing the OG

Done.

• line 324: please give the duration of the blotting step

We added the duration of the blotting step.

• line 345: please provide reference for CTER
• line 374: reference for Chimera
• line 377: references for COOT and PHENIX

We added the requested citations.

**Reviewer #3**: Although the author’s structural data are convincing and compelling, readers would greatly benefit from a revised discussion where the author’s lay out the evidence in the literature that would support the biological relevance of their findings. In particular, these and other structural studies have relied on interactions between PA and LF at micromolar or near micromolar concentrations which may be unlikely to occur during in vivo infection. What is the existing evidence that, in vivo, three substrates bind to the PA pore at nM or pM concentrations? Please discuss existing quantitative binding that indicates the affinity of these interactions.

We extended our discussion with the raised points.

In addition, the authors should discuss the direct experimental evidence that LF (or EF) are actually translocated when all three sites of PA are occupied? The authors should discuss existing data that indicate that all three PA sites be occupied for translocation, or do existing data indicate that LF/EF can be translocated regardless of whether 1, 2, or 3 effector binding sites on PA are occupied? What do existing data tell us about whether the second or third LF/EF to bind PA are actually ever translocated following translocation of the first molecule? In other words, what DO we currently know, and what do we NOT currently know about the items discussed above in relation to infection in vivo.

We included the requested points in our revised discussion.
